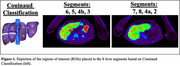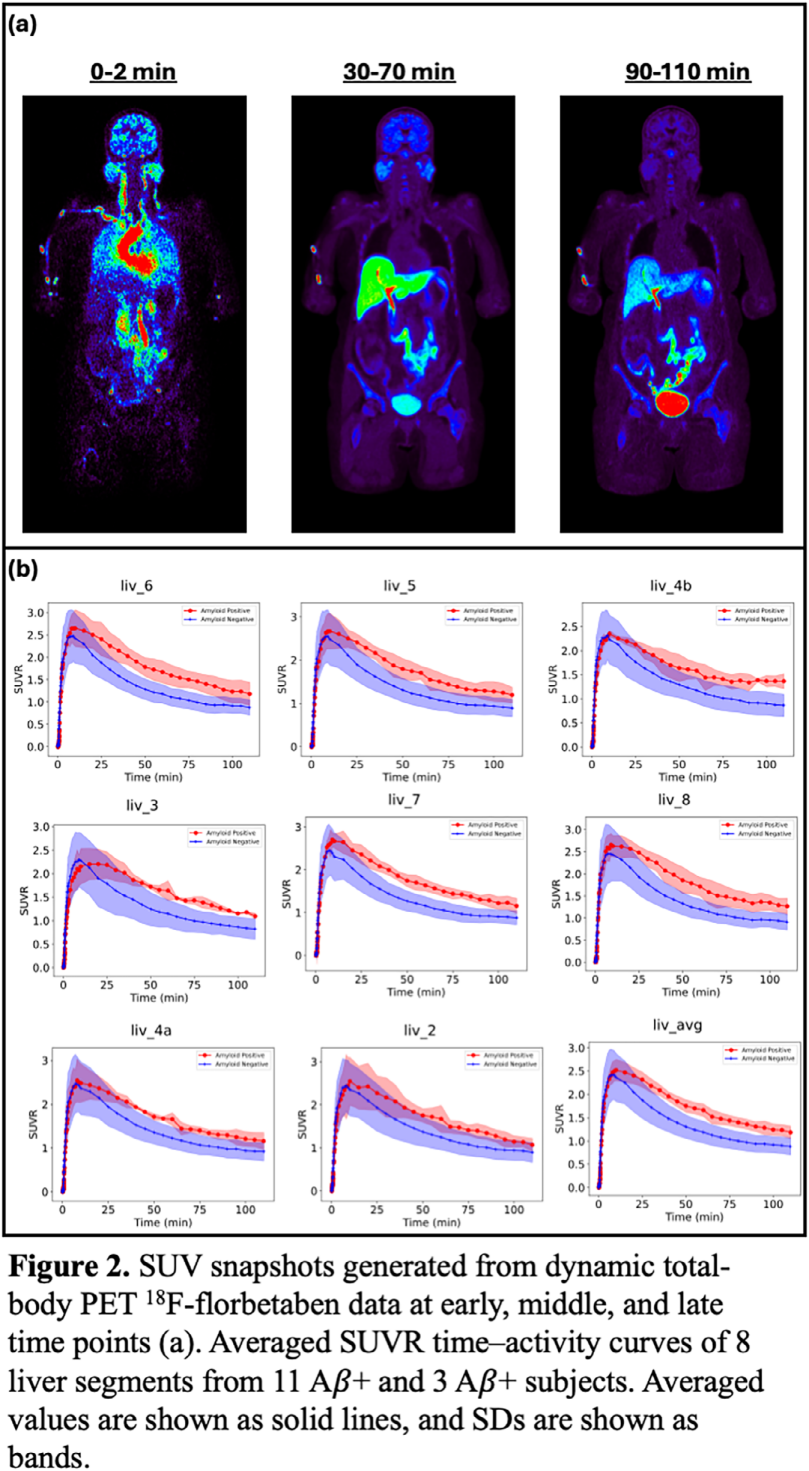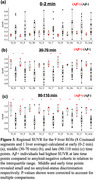# Total‐body PET Amyloid Signal in the Liver in Alzheimer's Disease

**DOI:** 10.1002/alz70856_104641

**Published:** 2025-12-26

**Authors:** Emily N. Holy, Guobao Wang, Benjamin A. Spencer, Simon R. Cherry, Charles Decarli, Audrey P. Fan

**Affiliations:** ^1^ University of California Davis, Davis, CA, USA; ^2^ Alzheimer's Disease Research Center, University of California Davis, Sacramento, CA, USA; ^3^ Department of Neurology, University of California, Davis, Davis, CA, USA

## Abstract

**Background:**

Although Alzheimer's is widely considered a “disease of the brain”, characterized by accumulation of amyloid‐beta (Aβ) plaques, Aβ is present and degraded not only in the brain but also in the blood and liver. Previous molecular studies have shown individuals with Alzheimer's disease have significantly different amyloid loads in the liver compared to healthy controls. Current PET imaging of amyloid deposition focuses only on the brain, the site of AD pathology manifestation. Additionally conventional PET scanners have a limited ∼20 cm field of view (FOV), enabling coverage for one organ at a time. This technical constraint limits information about peripheral organs and how the brain and body are critically linked in molecular processes. The aim of our study is to leverage the total‐body uEXPLORER PET to characterize amyloid signal over time and to highlight differences in liver Aβ with standard uptake value ratio (SUVR).

**Method:**

^18^F‐florbetaben dynamic PET imaging was performed on the uEXPLORER system with tracer injection (300 MBq) in 3 Aβ+ individuals with AD and 11 Aβ‐ individuals. Regions of interest (ROIs) were placed in the eight liver segments based on the Couinaud classification to account for liver heterogeniety (Figure 1). An additional composite liver ROI was created by averaging TACs from the 8 segments. SUVR time activity curves were generated over 90‐110 min. Linear mixed effects models were used to understand how A status and liver regions were associated with SUVR including a person‐specific random effect to account for multiple comparisons.

**Result:**

There was increased liver SUVR in the Aβ+ compared to the Aβ‐ group, with significant discrimination for all regions at later time points (*p* <0.05) (Figure 2b, 3c), little discrimination at middle time points (Figure 3b), and no discrimination at early time points (Figure 3a).

**Conclusion:**

Like the brain, separation of Aβ+ and Aβ‐ individuals over time occurred at later time points in the liver, suggesting 90‐110 min is the optimal window for amyloid signal evaluation in the liver. Current work in progress is building upon these findings by using simultaneous total‐body quantitative kinetic modeling in brain and liver to highlighting specific systemic molecular processes in AD.